# Single-Center Real-World Experience with Sutureless Aortic Valve Prosthesis in Isolated and Combined Procedures

**DOI:** 10.3390/jcm12124163

**Published:** 2023-06-20

**Authors:** Alina Zubarevich, Lukman Amanov, Arian Arjomandi Rad, Eleftherios T. Beltsios, Marcin Szczechowicz, Anja Osswald, Arjang Ruhparwar, Alexander Weymann

**Affiliations:** 1 Department of Thoracic and Cardiovascular Surgery, West German Heart and Vascular Center, University of Duisburg-Essen, University Hospital Essen, 45122 Essen, Germany; 2 Medical Sciences Division, University of Oxford, Oxford OX3 9DU, UK; 3 Department of Cardiothoracic, Transplant and Vascular Surgery, Hannover Medical School, 30625 Hannover, Germany

**Keywords:** sutureless aortic valve, SU-AVR, aortic valve replacement, Perceval

## Abstract

Introduction: Due to their favourable hemodynamic performance and the ability to enable minimally invasive access procedures, sutureless aortic valve prostheses have found their place in the armamentarium of cardiothoracic surgeons. In this study, we sought to review our institutional experience of sutureless aortic valve replacement (SU-AVR). Methods: We carried out a retrospective analysis of 200 consecutive patients who underwent an SU-AVR with a Perceval valve between December 2019 and February 2023. Results: The mean age of patients was 69.3 ± 8.1 years, and patients showed a moderate-risk profile with a mean logistic EuroSCORE-II of 5.2 ± 8.1%. An isolated SU-AVR was performed in 85 (42.5%) patients, concomitant CABG was performed in 75 (37.5%) and 40 patients (20%) underwent a multivalve procedure involving SU-AVR. The cardiopulmonary bypass (CPB) and cross-clamp (CC) times were 82.1 ± 35.1 and 55.5 ± 27.8 min, respectively. In-hospital, 30-day, 6-month and 1-year mortality rates were 4.5%, 6.5%, 7.5% and 8.2%, respectively. The postoperative transvalvular mean pressure gradient was 6.3 ± 1.6 mmHg and stayed stable over the follow-up time. We reported no cases of paravalvular leakage, and the incidence of stroke was 0.5%. Conclusions: With their favourable hemodynamic performance and shorter CC and CPB times, sutureless aortic valve prostheses facilitate minimally invasive access surgery, being a safe and durable promising approach for the surgical AVR.

## 1. Introduction

Surgical aortic valve replacement (SAVR) is one of the most commonly performed procedures in cardiac surgery, proven to be a definitive treatment that substantially improves the clinical symptoms and the long-term survival of patients. Having been considered the gold standard for more than half a century, hundreds of thousands of patients have benefited from this procedure, with complications and mortality continuously decreasing [1,2].

Despite the great variety of valve prostheses available on the market, the principles of SAVR remain unchanged. In essence, following the removal of the native diseased valve via extracorporeal circulation (ECC) and cross-clamping of the aorta, the new prothesis is anchored and sutured in the aortic annulus. A significant portion of the cross-clamping time is attributed to the placement and the tying of the knots for the sutures in the aortic annulus and the valve prosthesis [3]. A long cardiopulmonary bypass and aortic cross-clamp (CC) time are proven to be important independent risk factors of mortality in patients undergoing cardiovascular procedures [4]. As the population ages, patients present with more comorbidities such as diabetes, diffuse atherosclerosis, and higher rates of root and valve calcification [5]. To mitigate the risk of ischemia reperfusion injury during valve procedures, efforts are underway to reduce ECC and aortic CC time. Amongst the new approaches, sutureless aortic valve replacement (SU-AVR) and transcatheter aortic valve implantation (TAVI) are considered less invasive, alternative approaches for aortic valve patients [6,7,8].

The development of sutureless aortic valves was driven by the need to mitigate the significant challenges associated with traditional surgical valve replacements. Specifically, sutureless valves were designed to reduce cross-clamp and cardiopulmonary bypass times, as well as the overall operative time, in order to lower the risks associated with prolonged procedures [9,10,11,12]. In addition to these benefits, sutureless valves also aim to address some key limitations of stented valves. These include preserving the annulus distensibility, which can provide a larger orifice area, and resecting the native diseased valve to minimize paravalvular leakage [13,14].

A number of studies have reported satisfactory hemodynamic and clinical outcomes, while the operative time was found to be reduced compared to the traditional surgical approach [15,16,17,18,19]. Evidence from the Sutureless and Rapid-Deployment Aortic Valve Replacement International Registry further suggested that SU-AVR may provide improved hemodynamic results, while promoting minimal invasive surgery and achieving reduced CPB and cross-clamp time [20]. Sutureless valve replacement, especially when performed with minimally invasive approaches, offers an attractive option for patients eligible for biological valve replacement [21,22]. Nevertheless, the long-term outcomes and durability of sutureless valves demand further investigation.

The aims of our study encompass several crucial facets of sutureless aortic valve prosthesis application. Our primary goal is to offer a comprehensive report on real-world outcomes associated with the use of the Perceval prosthesis, utilizing the ‘Snugger Method’ of implantation mastered by our experienced surgical team which already mastered the learning curve. Concurrently, we aim to explore and document its use across a diverse range of indications, including those currently considered off-label in Europe, such as infective endocarditis and isolated aortic regurgitation. Moreover, we seek to provide an in-depth review of our experiences, lessons learned and practical guidance for avoiding procedural missteps, such as oversizing. Ultimately, our intention is to augment the existing body of knowledge by demonstrating the consistent application of implantation techniques over time, unveiling the potential of off-label indications and sharing real-world insights.

## 2. Materials and Methods

### 2.1. Study Design

The present study is a nonrandomized, retrospective, single-center study, including 200 consecutive patients who underwent a sutureless aortic valve replacement (SU-AVR) using the Perceval S valve at our institution between December 2019 and February 2023.

### 2.2. Data Collection

Information about patient demographics, clinical characteristics and operative data were retrospectively extracted from patient records, in accordance with the regulations on data protection. An active follow-up was performed via telephone interviews with the patients and/or their primary care physicians. A postoperative echocardiographic evaluation of the hemodynamic performance of the implanted valve prosthesis was performed prior to hospital discharge. The mean gradient was measured with transthoracic echocardiography one day prior to hospital discharge, while the follow-up gradient was the last measured gradient by the referring cardiologist at the control appointment, which were carried out every 3 to 6 months, depending on the stability of the valve performance.

The requirement for informed consent was deferred due to the observational, retrospective nature of the study. The present study was performed in accordance with the Declaration of Helsinki. The data regarding the patient identity remained strictly anonymous. Ethical approval was obtained prior to commencement of the present study from the Ethics Committee of the University Hospital of Essen, Essen, Germany (21-10349-BO). All methods were performed in accordance with the regulations and guidelines.

### 2.3. Inclusion Criteria

Every patient who underwent an SU-AVR using the Perceval valve with or without concomitant procedures was eligible for the study. Patients undergoing redo procedures, presenting with infective endocarditis or isolated aortic valve regurgitation were not seen as a contraindication for Perceval valve implantation, neither did patients presenting with bicuspid aortic valves or severely calcified ascending aorta. In patients presenting with severe aortic annulus dilatation and/or aneurysm of the ascending aorta, Perceval S prosthesis was not implanted. All cases were evaluated preoperatively by our institutional interdisciplinary Heart Team consisting of a cardiac surgeon, cardiac anesthesiologist and interventional cardiologist. The type of valve implemented in each case was individually chosen at the surgeons’ discretion, depending on the availability, and was always in accordance with the latest guidelines.

### 2.4. Outcomes and Definitions

The primary endpoints of this study were in-hospital, 30-day, 6-month and 1-year mortality and device success, which were evaluated with transthoracic echocardiography. The secondary endpoint was the development of any postoperative adverse events as defined by the Valve Academic Research Consortium (VARC-2) [23].

Urgent procedures were defined as procedures which had to be performed during the same in-hospital stay. Emergent procedures were defined as procedures which had to be performed within the next 24 h.

### 2.5. Statistical Analysis

The obtained data were entered into a dedicated Microsoft Excel spreadsheet. Statistical analysis was performed using IBM SPSS version 27 (IBM Corp., Chicago, IL, USA). Data were tested for normality using the Shapiro–Wilk test. When the data were not normally distributed, continuous variables were expressed as medians (interquartile range, IQR) or as mean ± standard deviation. Categorical variables were expressed as frequencies and percentages. A *p*-value of less than 0.05 was considered statistically significant. Survival curves were generated using the Kaplan–Meier method.

### 2.6. Surgical Techniques

In all cases, Perceval prosthesis was implanted using the ‘snugger method’, as previously described [24]. Concomitant procedures were combined with SU-AVR and performed as described in our recent research [7,11,25,26,27].

## 3. Results

A total of 200 consecutive patients with multiple comorbidities were enrolled in the study. The mean age was 69.3 ± 8.1 years. An isolated SU-AVR was performed in 85 (42.5%) patients, concomitant CABG was performed in 75 (37.5%) patients and 40 patients (20%) underwent a multivalve procedure involving an SU-AVR. In our cohort, we had sixteen patients (8%) who were suffering from active infective endocarditis, but showed no signs of an annular abscess and maintained an intact aortic annulus. The baseline characteristics and demographics are shown in Table 1. Overall, the patients showed a moderate-risk profile with a mean logistic EuroSCORE-II of 5.2 ± 8.1%. Eighteen patients (9%) had previously undergone open cardiac surgery via median sternotomy. A total of 43 patients (21.5%) showed a pre-operative impaired renal function.

### 3.1. Procedure

The majority of patients were treated via median sternotomy (96%), whereas 33 patients (16.5%) underwent a minimally invasive procedure. The implantation of the Perceval S prosthesis was technically successful in 99.5% of patients, without any intra-procedural complications. In one patient, we observed a case of Perceval dislocation, which was detected during intraoperative echocardiography and corrected instantly. No left ventricular outflow tract obstruction occurred, and no second valve was required (Table 2). The overall procedural time (skin-to-skin) was relatively short, averaging 138.1 ± 46.4 min, and the mean CPB time was 82.1 ± 35.1 min and the CC time for all procedures was 55.5 ± 27.8 min.

### 3.2. Postoperative Outcomes and Survival

**Figure 1:**
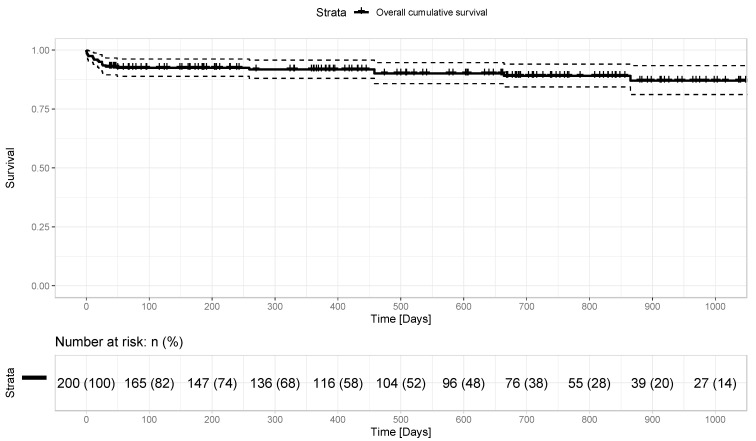
Overall survival.

The mean follow-up time for the whole cohort was 549.5 ± 465.0 days. The follow-up in the present study has been complete. A total of 14 patients (7%) required re-exploration for bleeding, and another 8 patients (4%) suffered postoperative AV-Block III°, requiring permanent pacemaker implantation. We observed no postoperative myocardial infarction and just one case of stroke (0.5%) in our cohort. There were no reported cases of recurrent valve endocarditis among the patients initially diagnosed with active endocarditis. Acute kidney injury requiring temporary dialysis occurred in 17 (8.5%) patients. The early postoperative aortic valve mean pressure gradient was 6.3 ± 1.6 mmHg, which remained stable over the follow-up period at 5.9 ± 2.2 mmHg. The mean time from the hospital discharge to the last echocardiographic evaluation was 503.53 ± 455.8 days. The mean in-hospital stay was 9.1 ± 4.6 days and there were no cases of intraprocedural death within the whole cohort. The in-hospital, 30-day, 6-month and 1-year mortality rates are presented in the Table 3. The overall survival at follow-up is portrayed in Figure 1. There was just one case of an early re-operation due to infective endocarditis at follow-up.

## 4. Discussion

In this single-center study, we offer a unique perspective on sutureless prosthesis application, guided by the seasoned proficiency of our surgical team that already mastered the learning curve. Earlier studies have largely focused on the early stages of Perceval implantation, a time of rapid procedural evolution, and associated complications. Our research, however, centers on a cohort treated by a team that has mastered the ‘Snugger Method’ of implantation, yielding consistently favorable outcomes since its establishment in 2017. Moreover, our study explores Perceval prosthesis application across a range of indications, including those considered off-label in Europe, such as infective endocarditis, multivalve disease and isolated aortic regurgitation, all of which we have already described in our previous research suggesting Perceval’s feasibility in all the alternative indications [11,25,27,28]. Our real-world cohort, informed by the practical wisdom of centers globally, presents a unique opportunity to learn from their experiences and avoid early missteps like oversizing. Thus, our research not only bolsters existing knowledge, but also introduces fresh insights by showcasing consistent techniques, revealing potential off-label uses and sharing experienced team insights.

The growing population of elderly patients suffering from aortic valve disease highlights the importance of implementing minimally invasive techniques that can reduce ischemia reperfusion injury during aortic valve replacement [5]. In this regard, SU-AVR and TAVI are both considered promising and less invasive approaches for these patients [6,7,8]. Although the use of TAVI in the lower-risk patients is increasingly supported by evidence in the literature [29], most of the studies which compare TAVI with the standard surgical AVR do not include patients who underwent an SU-AVR. Only a limited number of studies that compared TAVI with surgical AVR performed with sutureless valves are available in the literature. In the present study, a total of 200 intermediate-risk patients presenting with moderate-to-severe aortic valve disease were treated with sutureless valve prosthesis as either an isolated or combined procedure.

The Perceval valves, which require only three temporary guiding sutures, involve limited manipulation in the aortic root and result in a substantially shorter implantation time. This is expected to yield several clinical benefits for patients, including a shorter hospital stay and ICU stay, and low rates of 30-day mortality, stroke and other adverse events [30]. Although Perceval valves facilitate less invasive approaches in patients with isolated AVR, in our study, only 16.5% of the patients were operated through a minimal invasive access, which is lower than the current worldwide adoption (approximately 20 to 25%). This could be explained by the increasing learning curve of the surgeons preforming the procedures. As our center gained more experience with SU-AVR over the last year, we observed a definite increase in isolated SU-AVR via RALT or J-sternotomy. The same effect has also been demonstrated in the Sutureless and Rapid-Deployment Aortic Valve Replacement International Registry.

Mortality, as well as a number of severe complications, is commonly associated to cross-clamp and CPB time in the literature [31,32]. Sutureless aortic valve prostheses facilitate reduction in CPB, cross-clamp and total procedural time, promising favorable morbidity and mortality rates. In fact, in our recently published study, a significant reduction in the procedural time was shown in the sutureless group when compared to the standard surgical AVR group [33] within the context of multivalve procedures [28]. In this study, total procedural, CPB and cross-clamp times were found to be comparable to previously published studies [30,34]. A low rate of procedural mortality was previously reported [30], while there were no cases of intraprocedural death in the whole cohort in our study.

Our study presents an overall low rate of postoperative complications and ICU- and in-hospital lengths of stay, comparable to previously published studies [30]. The incidence of stroke was 0.5%, we reported no postoperative myocardial infarction and 17 patients (8.5%) presented acute kidney injury requiring temporary dialysis in our cohort.

Our results further confirm the previously reported low 30-day mortality for our heterogenous cohort [25], while also presenting 6-month and 1-year mortality rates of 7.5% and 8.2%, respectively, for the whole cohort. These relatively high mortality rates may be explained by the significantly higher mortality of the multivalve procedure group which consists of patients carrying a higher operative risk compared to Groups 1 and 2. None of the deaths were procedure- or valve-prosthesis-related. In our earlier research, we have described the relatively high mortality in the multivalve group due to the higher operative risk portrayed by the EuroSCORE II [25].

The previously described hemodynamic results and low rate of paravalvular leakage [30] are in line with the results of the present study. The early postoperative aortic valve mean pressure gradient was found to be 6.3 ± 1.6 mm Hg in our cohort, which remained stable over the follow-up period, at 5.9 ± 2.2 mm Hg.

There has been a discussion in the literature regarding pacemaker implantation rates in patients who underwent SU-AVR, which appeared to be higher when compared to conventional aortic valve prostheses in early evidence [35,36]. The results from multiple larger studies indicated that pacemaker implantation rates are significantly lower at high-volume reference centers [20,37], mainly due to the association between the learning curve of the surgeon and the pacemaker implantation rates [38,39]. Appropriate sizing, and most importantly the avoidance of oversizing, is of high importance with regards to the reduction in postoperative complications. It has been shown, that correct sizing is associated with reduced peak and mean gradients at discharge, less paravalvular leakage and lower permanent pacemaker implantation rates [30,40,41,42]. In fact, in this study, we reported no paravalvular leakage in our results and low rates of permanent pacemaker implantation. Particularly, we reported an overall 30-day pacemaker implantation rate of 4%, of which 1.5% in isolated AVR, 0.5% in AVR combined with CABG and 2% in multivalve procedures, respectively.

Aortic-valve-related re-operations in patients who underwent SU-AVR are rarely reported in the literature [43]. Our study further supports these results, since we report only one case of an early re-operation due to infective endocarditis at follow-up in our cohort. Studies presenting data with longer follow-up times indicate that the freedom of AV-related reintervention remains high at long term, while in cases with degeneration of the valve, a transcatheter reintervention is a safe and effective solution for degenerated Perceval valves [30,44].

## 5. Limitations

There are several limitations in the present study. It is a single-center study with a relatively small sample size, and has a retrospective design. Another limitation is the absence of control groups such as patients receiving conventional AVR with sutured aortic valves. Moreover, the follow-up time was limited to less than 2 years. A longer follow-up time is crucial to provide stronger evidence for the long-term results. Further investigation, preferably through large-scale multicenter studies with longer follow-up time periods, is necessary to shed more light on the topic, providing stronger evidence.

## 6. Conclusions

To share our center’s experience and investigate the early outcomes of SU-AVR with Perceval prosthesis, we retrospectively analyzed the data of 200 consecutive patients who underwent SU-AVR at our institution. Although, there are several larger studies on the implementation of sutureless aortic valve prostheses, this study contributes the real-world patient cohort, in which sutureless prostheses have been implanted in all indications, while they are still to be officially expanded to the pure aortic regurgitation, redo and multivalve procedures in Europe. All patients in our cohort have been treated by a surgical team, in which the surgeons have fully completed the learning curve, so the perioperative results seem to have reached a steady state, not differing between the beginning and the end-phase of the observational period. We presented favorable early outcomes, relatively low rates of mortality in isolated and AVR + CABG groups, and low major complications rates. We report no cases of paravalvular leakage and low rates of permanent pacemaker implantation, highlighting the importance of correct sizing. Perceval valve prosthesis facilitates shorter cross-clamp and CPB times, being a safe and durable emerging approach for surgical AVR. With their excellent hemodynamic performance, Perceval sutureless aortic valve prostheses present feasible alternatives for conventional AVR, allowing a no- or J-sternotomy minimally invasive access.

## Figures and Tables

**Table 1:** Baseline characteristics and demographics.

Clinical Variable	Overall (*n* = 200)	Isolated AVR (*n* = 85)	AVR + CABG (*n* = 75)	Multivalve Procedures (*n* = 40)
Age, years	69.3 ± 8.1	66.6 ± 7.5	71.2 ± 7.3	71.5 ± 9.3
Female sex	73 (36.5%)	34 (17%)	19 (9.5%)	20 (10%)
BMI, kg/qm	28.4 ± 5.7	28.9 ± 6.0	28.4 ± 5.5	27.1 ± 5.6
Previous cardiac surgery	18 (9%)	12 (6%)	1 (0.5%)	5 (2.5%)
Diabetes mellitus type 2	61 (30.5%)	24 (12%)	26 (13%)	11 (5.5%)
Arterial hypertension	183 (91.5%)	73 (36.5%)	73 (36.5%)	37 (18.5%)
Pulmonal hypertension (sysPAP > 31 mmHg)	36 (18%)	8 (4%)	14 (7%)	14 (7%)
Cronic obstructive lung disease	31 (15.5%)	11 (5.5%)	13 (6.5%)	7 (3.5%)
Previous stroke	12 (6%)	6 (3%)	3 (1.5%)	3 (1.5%)
Coronary arterial disease	122 (61%)	30 (15%)	75 (37.5%)	17 (8.5%)
Previous PCI	31 (15.5%)	11 (5.5%)	14 (7%)	6 (3%)
Atrial fibrillation	47 (23.5%)	18 (9%)	14 (7%)	15 (7.5%)
Permanent	13 (6.5%)	4 (2%)	4 (2%)	5 (2.5%)
Paroxysmal	34 (17%)	14 (7%)	10 (5%)	10 (5%)
Kidney function impairment	43 (21.5%)	16 (8%)	14 (7%)	13 (6.5%)
Creatinine, g/dL	1.3 ± 1.2	1.2 ± 1.1	1.4 ± 1.3	1.5 ± 1.3
EuroSCORE II, %	5.2 ± 8.1	1.9 (IQR 2.4–5.0)	2.7 (IQR 2.8–4.1)	5.7 (IQR 7.2–16.0)
Urgency				
Elective	163 (81.5%)	70 (35%)	64 (32%)	26 (13%)
Urgent	28 (14%)	11 (5.5%)	9 (4.5%)	8 (4%)
Emergent	9 (4.5%)	2 (1%)	2 (1%)	5 (2.5%)
EF, %	53.2 ± 9.5	54.9 ± 10.1	53.2 ± 8.6	49.3 ± 8.9
Aortic stenosis > II°	163 (81.5%)	69 (34.5%)	71 (35.5%)	23 (11.5%)
Aortic regurgitation > II°	42 (21%)	18 (9%)	8 (4%)	16 (8%)
AV-mean pressure gradient, mmHg	42.2 ± 15.7	43.9 ± 15.2	44.4 ± 15.5	34.4 ± 15.7
Mitral stenosis > II°	16 (8%)	1 (0.5%)	4 (2%)	11 (5.5%)
Mitral regurgitation > II°	30 (15%)	0	0	30 (15%)
Tricuspidal regurgitation >II°	10 (5%)	1 (0.5%)	0	9 (4.5%)
TAPSE, mm	21.9 ± 3.0	22.3 ± 3.1	22.4 ± 2.3	20.0 ± 3.3

AV—aortic valve, BMI—Body Mass Index, EF—ejection fraction, sysPAP—systolic pulmonary arterial pressure, PCI—percutaneous coronary intervention, TAPSE—tricuspid annular plane systolic excursion.

**Table 2:** Intraoperative data.

Variable	Overall (*n* = 200)	Isolated AVR (*n* = 85)	AVR + CABG (*n* = 75)	Multivalve Procedures (*n* = 40)
Median sternotomy	167 (83.5%)	52 (26%)	75 (37.5%)	40 (20%)
Minimally invasive approach	33 (16.5%)	33 (16.5%)	0	0
J-sternotomy	21 (10.5%)	21 (10.5%)	0	0
RALT	12 (6%)	34 (17%)	19 (9.5%)	20 (10%)
Perceval size				
S	15 (7.5%)	3 (1.5%)	6 (3%)	6 (3%)
M	44 (22%)	19 (9.5%)	10 (5%)	15 (7.5%)
L	75 (37.5%)	30 (15%)	32 (16%)	13 (6.5%)
XL	66 (33%)	33 (16.5%)	27 (13.5%)	6 (3%)
Operating time, min	138.1 ± 46.4	111.4 ± 34.7	152.1 ± 43.7	168.4 ± 43.4
CPB time, min	82.1 ± 35.1	63.5 ± 25.3	86.9 ± 29.0	112.8 ± 39.0
CC time, min	55.5 ± 27.8	39.4 ± 17.8	59.5 ± 18.9	82.0 ± 35.5
Implant dislocation	1 (0.5%)	1 (0.5%)	0	0
Intraoperative blood transfusion, U	1.7 ± 1.8	0 (IQR 0.8–1.5)	2 (IQR 1.3–2.0)	2 (IQR 2.0–3.4)
Concomitant procedure				
CABG	85 (42.5%)	0	74 (37%)	11 (5.5%)
LAA closure	8 (4%)	1 (0.5%)	4 (2%)	3 (1.5%)
TVR	11 (5.5%)	0	0	11 (5.5%)
MV replacement	20 (10%)	0	0	20 (10%)
MV repair	20 (10%)	0	0	20 (10%)
Myectomy	15 (7.5%)	8 (4%)	4 (2%)	3 (1.5%)

CABG—coronary artery bypass grafting, CC—cross clamp, CPB—cardiopulmonary bypass, LAA—left atrial appendage, MV—mitral valve, TVR—tricuspid valve repair.

**Table 3:** Postoperative outcomes and survival.

Variable	Overall (*n* = 200)	Isolated AVR (*n* = 85)	AVR + CABG (*n* = 75)	Multivalve Procedures (*n* = 40)
ICU length of stay, days	2 (IQR 1–4)	2 (IQR 2.0–3.4)	2 (IQR 2.5–4.9)	3 (IQR 3.1–6.9)
In-hospital stay, days	9.1 ± 4.6	8.8 ± 4.0	9.5 ± 4.5	8.9 ± 5.9
Time on ventilator, days	1 (IQR 0.5–1)	1 (IQR 0.7–1.7)	1 (IQR 0.9–3.0)	1 (IQR 1.0–4.2)
procedural death	0	0	0	0
In-hospital death	9 (4.5%)	0	1 (0.5%)	8 (4%)
30-day mortality	6.5%	2.4%	4%	20%
6-month mortality	7.5%	2.4%	4%	25%
1-year mortality	8.2%	4%	4%	25%
Pacemaker at 30 days	8 (4%)	3 (1.5%)	1 (0.5%)	4 (2%)
Exploration for bleeding	14 (7%)	7 (3.5%)	5 (2.5%)	2 (1%)
Stroke/TIA	1 (0.5%)	0	1 (0.5%)	0
New-onset dialysis	17 (8.5%)	3 (1.5%)	5 (2.5%)	9 (4.5%)
AV-MPG at discharge, mmHg	6.3 ± 1.6	6.2 ± 1.8	6.2 ± 1.5	6.6 ± 1.1
PVL at discharge	0	0	0	0
AV-MPG at follow-up, mmHg	5.9 ± 2.2	6.2 ± 1.9	5.9 ± 2.0	5.3 ± 2.9
PVL at follow-up	0	0	0	0
LVF at follow-up				
Normal (EF > 50%)	137 (68.5%)	68 (34%)	50 (25%)	19 (9.5%)
Moderate (EF 31–50%)	52 (26%)	11 (5.5%)	23 (11.5%)	18 (9%)
Poor (EF < 30%)	11 (5.5%)	6 (3%)	2 (1%)	3 (1.5%)
Re-operation at follow-up	1(0.5%)	1 (0.5%)	0	0
Endocarditis at follow-up	1(0.5%)	1 (0.5%)	0	0
Follow-up time, days	549.5 ± 465.0	529.6 ± 348.3	577.5 ± 593.3	539.3 ± 414.4

AV—aortic valve, ICU—intensive care unit, LVF—left ventricular function, MPG—mean pressure gradient, TIA—transitory ischemic attack, PVL—paravalvular leakage.

## Data Availability

The data that support the findings of this study are available from the corresponding author upon reasonable request.
